# Association of plasma biomarkers with longitudinal change in age- and Alzheimer’s disease-related brain atrophy patterns

**DOI:** 10.64898/2026.09.11.26362869

**Published:** 2026-09-17

**Authors:** Murat Bilgel, Ishaan Shah, Jasmine Cooper, Yang An, Keenan A. Walker, Sara G. Ho, Abhay R. Moghekar, Zhijian Yang, Guray Erus, Christos Davatzikos, Luigi Ferrucci, Susan M. Resnick

## Abstract

**INTRODUCTION:**

Determining whether plasma biomarkers are preferentially associated with Alzheimer’s disease (AD)-related rather than age-related brain atrophy patterns may clarify their prognostic and diagnostic clinical use.

**METHODS:**

Using data from the Baltimore Longitudinal Study of Aging ( *N* =818), we examined cross-sectional plasma Aβ _42_ /Aβ _40_ , GFAP, NfL, p-tau181, and p-tau217 measurements obtained while participants were cognitively unimpaired (CU). During follow-up, 104 participants developed mild cognitive impairment (MCI)/dementia (74 due to AD, 24 due to non-AD, 6 unknown etiology). 2,293 longitudinal brain MRIs were used to quantify multidimensional atrophy pattern scores reflecting brain age (SPARE-BA), AD-like patterns (SPARE-AD), and five dominant dimensions of atrophy (R-indices). We investigated the associations of plasma biomarkers and atrophy pattern scores at index visit with conversion to MCI/dementia due to AD. We then examined the associations of plasma biomarkers with longitudinal change in pattern scores using linear mixed effects models.

**RESULTS:**

p-tau181, Aβ _42_ /Aβ _40_ (Lumipulse), and p-tau217 were associated with incident MCI/dementia due to AD but not non-AD etiologies, while GFAP was associated with incident MCI/dementia due to both AD and non-AD. All four biomarkers were associated with longitudinal SPARE-AD changes. Aβ _42_ /Aβ _40_ (Quanterix and Lumipulse), p-tau181, and p-tau217 were associated with longitudinal parieto-temporal atrophy. p-tau217 was the only biomarker associated with longitudinal medial temporal lobe atrophy. We did not find associations between plasma biomarkers and SPARE-BA or R-indices capturing subcortical, diffuse cortical, or perisylvian atrophy.

**DISCUSSION:**

Among CU individuals, plasma p-tau217 was associated with subsequent MCI/dementia due to AD and showed the most extensive associations with longitudinal AD-related brain atrophy.

## Full Text Availability

The license terms selected by the author(s) for this preprint version do not permit archiving in PMC. The full text is available from the preprint server.

